# Association of the HIGS score with permanent pacemaker implantation and mortality after transcatheter aortic valve implantation

**DOI:** 10.1080/07853890.2026.2715272

**Published:** 2026-08-11

**Authors:** Özkan Karaca, Burak Toprak, Nihat Söylemez, Mustafa Ekici, Abdulkadir Bilgiç, Ahmet Turhan Kılıç, Samet Yımaz, Ali Orçun Sürmeli, Sonay Oğuz, Mehmet Ballı, Rıdvan Bora, Serdar Keçeoğlu

**Affiliations:** ^a^ Department of Cardiology, Mersin City Education and Research Hospital, Toroslar/Mersin, Turkey; ^b^ Department of Cardiovascular Surgery, Mersin City Education and Research Hospital, Toroslar/Mersin, Turkey; ^c^ Department of Emergency Medicine, Mersin Provincial Health Directorate, Akdeniz/Mersin; ^d^ Department of Cardiovascular Surgery, Mersin University Faculty of Medicine Hospital, Yenişehir-Mersin, Turkey; ^e^ Department of Cardiology, Başkent University Adana Research Center, Adana, Turkey

**Keywords:** Transcatheter aortic valve implantation, permanent pacemaker implantation, HIGS score, hematological inflammatory markers, ınflammation, mortality, risk stratification

## Abstract

**Background:**

Permanent pacemaker implantation (PPI) and early mortality remain important complications after transcatheter aortic valve implantation (TAVI). The Hematological Inflammatory Global Score (HIGS), derived from routine hematological parameters, may provide prognostic information after TAVI. This study evaluated the association of HIGS with PPI and in-hospital mortality.

**Methods:**

This retrospective single-center study included 196 consecutive patients undergoing TAVI (mean age 78.2 ± 6.9 years; 71.4% male). HIGS was calculated using standardized values of red cell distribution width, platelet distribution width, and immature granulocyte percentage. Associations with outcomes were assessed using logistic regression, and discrimination was evaluated by receiver operating characteristic analysis.

**Results:**

PPI occurred in 43 patients (21.9%) and in-hospital mortality in 34 (17.3%). HIGS was higher in patients with PPI and in those who died (both *p* < 0.001). In multivariable analysis, HIGS remained independently associated with PPI (OR 3.96, 95% CI 1.79–8.76, *p* < 0.001) and mortality (OR 4.92, 95% CI 2.12–11.41, *p* < 0.001). HIGS showed good discrimination for PPI (AUC 0.84) and mortality (AUC 0.87), and improved conventional clinical models.

**Conclusions:**

Higher HIGS values were independently associated with PPI and in-hospital mortality after TAVI. HIGS may be a simple, readily available risk-stratification marker, although prospective multicenter validation is required.

## GRAPHİCAL ABSTRACT

**Figure:**
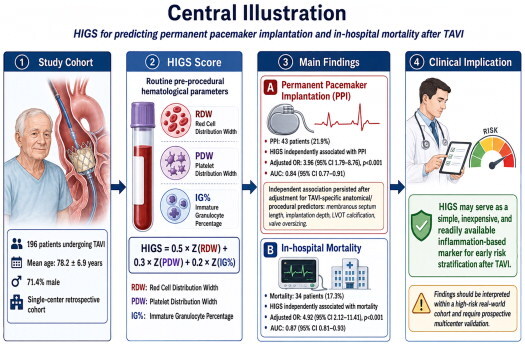

**Central Illustration:** Overview of the study design, HIGS score calculation, main clinical findings, and potential clinical implications of HIGS in patients undergoing transcatheter aortic valve implantation. HIGS was calculated using standardized values of RDW, PDW, and IG% and was independently associated with both permanent pacemaker implantation and in-hospital mortality. The association between HIGS and PPI persisted after adjustment for TAVI-specific anatomical and procedural predictors, including membranous septum length, implantation depth, LVOT calcification, and valve oversizing. HIGS = Hematological Inflammatory Global Score; TAVI = Transcatheter Aortic Valve Implantation; PPI = Permanent Pacemaker Implantation; RDW = Red Cell Distribution Width; PDW = Platelet Distribution Width; IG% = Immature Granulocyte Percentage; LVOT = Left Ventricular Outflow Tract; AUC = Area Under the Curve; OR = Odds Ratio; CI = Confidence Interval.

## Introduction

Aortic stenosis is one of the most common valvular diseases, particularly in the elderly population, and its incidence is steadily increasing in aging societies [1]. Severe symptomatic aortic stenosis is associated with high mortality when left untreated; therefore, effective and timely intervention is of great importance [2]. Although surgical aortic valve replacement had long been accepted as the standard treatment for this disease, alternative treatment strategies have been required, especially for patients with high surgical risk or those considered unsuitable for surgery [3].

Transcatheter aortic valve implantation (TAVI) has led to a major paradigm shift in the treatment of aortic stenosis in recent years. Initially performed in patients with high surgical risk, it is now increasingly used in intermediate- and even low-risk patient populations [4]. Randomized trials and large registry data have demonstrated that TAVI can provide clinical outcomes comparable to surgery, and in some cases even superior outcomes, in appropriately selected patient groups [4]. As a result of these developments, TAVI has become an important therapeutic option, particularly in elderly patients and those with multiple comorbidities [5]. It should also be recognized that real-world TAVI populations may substantially differ across centers according to patient selection strategies, procedural era, and baseline surgical risk profiles. In particular, cohorts consisting predominantly of elderly patients with multiple comorbidities and high surgical risk scores may demonstrate higher rates of adverse clinical outcomes compared with contemporary low-risk TAVI registries.

However, the TAVI procedure is associated with certain specific complications. One of the most notable is conduction system injury leading to the need for permanent pacemaker implantation (PPI) [6,7]. Because the aortic valve is anatomically located in close proximity to the atrioventricular conduction system, mechanical compression or trauma during valve implantation may result in conduction disturbances [7]. In addition to direct mechanical compression, several anatomical factors have been shown to influence the risk of conduction disturbances following TAVI. In particular, the length of the membranous septum, implantation depth of the valve prosthesis, and the spatial relationship between the prosthetic valve and the conduction system are recognized as important determinants of post-procedural conduction abnormalities. Previous imaging studies have demonstrated that shorter membranous septum length and deeper valve implantation are associated with an increased likelihood of permanent pacemaker implantation after TAVI [6–8]. Various studies have reported that the requirement for permanent pacemaker implantation after TAVI ranges between 5% and 25% [7,8]. The need for PPI is not only a procedure-related complication but may also have negative effects on long-term cardiac function, length of hospital stay, and mortality [8].

Identifying reliable risk markers that can predict mortality and the need for PPI after TAVI is of great importance for patient selection, procedural planning, and perioperative management. Although clinical characteristics, electrocardiographic findings, and procedural factors are commonly used to predict these risks, the prognostic value of inflammatory and hematological biomarkers has increasingly attracted attention [9]. Inflammation plays a significant role in the pathophysiology of cardiovascular diseases, and particularly in elderly patients, inflammatory activity may influence both procedural complications and clinical outcomes [10].

In recent years, several inflammatory scores derived from neutrophils, lymphocytes, and other hematological parameters have been shown to have prognostic value in cardiovascular diseases. Several hematological indices such as the neutrophil-to-lymphocyte ratio, platelet-to-lymphocyte ratio, and systemic inflammatory index have previously been investigated as prognostic markers in cardiovascular interventions. However, most of these markers evaluate only limited aspects of the inflammatory response. Composite indices that simultaneously reflect erythrocyte heterogeneity, platelet activation, and acute inflammatory response may provide a more comprehensive assessment of the systemic inflammatory state. In this context, the Hematological Inflammatory Global Score (HIGS) has emerged as a novel risk marker that comprehensively evaluates systemic inflammatory response and hematological alterations [11]. The HIGS score is considered a potential biomarker for predicting adverse outcomes in patients undergoing cardiovascular interventions and cardiac surgical procedures [11].

The HIGS score is a composite biomarker created by combining three fundamental hematological parameters obtained from routine complete blood count analysis. This score is based on the combined assessment of red cell distribution width (RDW), which reflects erythrocyte heterogeneity; platelet distribution width (PDW), which indicates platelet activation and thrombotic potential; and immature granulocyte percentage (IG%), which is considered a marker of acute inflammatory response [11]. Each of these parameters represents distinct biological processes involved in the pathophysiology of cardiovascular diseases.

RDW has been associated with inflammation, oxidative stress, and adverse cardiovascular outcomes, whereas PDW reflects platelet activation and thrombotic activity. IG% is considered a marker of acute inflammatory response and bone marrow activation. The combined assessment of these parameters may therefore provide a broader representation of systemic inflammatory and hematological alterations associated with cardiovascular complications [11–15]. One of the most important advantages of HIGS is that it is based on parameters easily obtained from routine laboratory tests, does not require additional cost, and can be rapidly calculated in clinical practice [11].

Despite the growing interest in inflammation-based hematological indices, studies specifically evaluating the association between the HIGS score and clinical outcomes after TAVI remain limited. Therefore, there remains a need for simple, accessible, and cost-effective risk markers to predict early clinical outcomes after TAVI.

The aim of this study was to evaluate the role of the Hematological Inflammatory Global Score (HIGS) in predicting permanent pacemaker implantation and mortality in patients undergoing transcatheter aortic valve implantation. Additionally, we evaluated whether the addition of the HIGS score to conventional clinical, electrocardiographic, and procedural variables could improve discrimination of adverse outcomes after TAVI.

## Materials and methods

### a. Data collection

#### Study design

This study was designed as a retrospective, observational, single-center cohort study to evaluate the prognostic value of the Hematological Inflammatory Global Score (HIGS) in predicting the need for permanent pacemaker implantation and mortality in patients undergoing transcatheter aortic valve implantation (TAVI). The study was conducted using data from consecutive patients who underwent the TAVI procedure in the Cardiology and Cardiovascular Surgery departments of Mersin City Training and Research Hospital. The research was based on real-world data and was carried out using electronic patient records and the hospital database obtained during routine clinical practice.

The study population included patients who underwent transcatheter aortic valve implantation at our center between September 2023 and November 2024. This study was designed as a retrospective observational cohort, and all eligible patients were included chronologically to minimize selection bias and reflect real-world clinical practice. The study was conducted and reported in accordance with the Strengthening the Reporting of Observational Studies in Epidemiology (STROBE) guidelines. Clinical, laboratory, and procedural data were retrospectively retrieved from the institutional electronic database between January 2026 and February 2026, during which data verification and statistical analyses were also performed.

The primary endpoints of the study were defined as in-hospital all-cause mortality following TAVI and the requirement for permanent pacemaker implantation due to persistent conduction disturbances after the procedure.

To obtain a relatively homogeneous study population, patients with conditions potentially affecting hematological parameters were not included. Patients with missing clinical data, those with active infections, hematological malignancies, or a history of immunosuppressive therapy that could affect hematological parameters were excluded from the analysis. In addition, individuals with acute systemic conditions that could significantly alter inflammatory parameters were not included in the study.

#### Data collection

The data evaluated within the scope of the study were collected under four main categories: demographic and clinical characteristics, laboratory parameters, electrocardiographic and echocardiographic findings, and procedural/anatomical TAVI-specific variables. All data included in the study were obtained retrospectively from the hospital information management system, electronic patient records, and procedural databases. During data collection and analysis, patient identity information was anonymized, and all data were used solely for scientific purposes.

The inclusion criteria for the study were as follows: undergoing the TAVI procedure based on the heart team decision with a diagnosis of symptomatic severe aortic stenosis, having complete pre-procedural clinical and laboratory data available, and having accessible post-procedural clinical follow-up data. All patients meeting these criteria were included in the study population.

To ensure the homogeneity of the study population and reduce potential confounding factors that could affect hematological parameters, specific exclusion criteria were applied. Patients with active infections, individuals with a history of hematological malignancies, patients receiving immunosuppressive therapy or systemic corticosteroid treatment, individuals with acute inflammatory diseases, and patients with systemic conditions that could significantly affect hematological parameters were excluded from the study. In addition, patients whose baseline laboratory data prior to the procedure were unavailable or whose clinical records were incomplete were also excluded from the analysis.

Demographic data included patient age and sex. Clinical data included comorbid conditions such as diabetes mellitus, hypertension, COPD, atrial fibrillation, and other relevant cardiovascular comorbidities. In addition, electrocardiographic parameters including QRS duration, PR interval, right bundle branch block, echocardiographic findings including left ventricular ejection fraction and aortic valve measurements, as well as HbA1c levels were included among the analyzed variables.

Additional anatomical and procedural variables relevant to post-TAVI conduction disturbances were collected and analyzed. These variables included membranous septum length, implantation depth, annular calcification grade, LVOT calcification grade, and valve oversizing percentage. Membranous septum length, annular calcification, and LVOT calcification were assessed from pre-procedural imaging and procedural planning records, whereas implantation depth and valve oversizing were obtained from procedural documentation and valve-sizing data. Annular and LVOT calcification were graded using an ordinal scale from 0 to 3, with higher grades indicating greater calcification burden. These variables were included in both comparative analyses and regression models to account for established anatomical and procedural determinants of post-TAVI conduction system injury.

Biochemical and hematological parameters were obtained as part of routine laboratory evaluations performed prior to the procedure. All laboratory samples were collected from venous blood samples obtained before the procedure according to standard clinical practice. Hematological parameters were derived from complete blood count analyses, and RDW, PDW, and IG% values constituted the primary biomarkers of the study. These parameters were measured using automated hematology analyzers, and analyses were conducted in accordance with the quality control procedures recommended by the manufacturer.

In addition, urea and creatinine levels were included in the dataset as indicators of renal function. These parameters are well-known biochemical markers closely associated with mortality in patients undergoing cardiovascular surgery and interventional cardiology procedures. Accordingly, creatinine and urea values were also evaluated as potential confounding variables in regression analyses.

All collected data were independently reviewed by two investigators, and cross-verification was performed to ensure data accuracy. Only patients with complete baseline clinical and laboratory data were included in the final analysis. A complete-case analysis approach was used, and no data imputation was performed.

### b. Data analysis

#### Statistical analysis

Statistical analyses were performed in accordance with the predefined analysis plan established at the beginning of the study. All analyses were conducted using two-sided hypothesis testing, and a *p* value < 0.05 was considered statistically significant.

Continuous variables were tested for normality using the Kolmogorov–Smirnov test and Q–Q plots. Normally distributed variables were expressed as mean ± standard deviation, whereas non-normally distributed variables were presented as median (minimum–maximum). Categorical variables were expressed as frequencies and percentages.

For comparisons between groups, Student’s t-test was used for normally distributed continuous variables. The Mann–Whitney U test was preferred for continuous variables that did not follow a normal distribution. Categorical variables were compared using the chi-square test, and Fisher’s exact test was used when the expected frequencies were low.

#### HIGS score calculation

One of the primary analyses of the study was the calculation of the HIGS score. The HIGS score was calculated using standardized z-scores of RDW, PDW, and IG% parameters. Standardization was performed using the mean and standard deviation values of each parameter within the study population. This approach allows biomarkers measured on different scales to be combined into a single composite score. The final score was calculated using the following formula:

(1) [HIGS=0.5×Z(RDW)+0.3×Z(PDW)+0.2×Z(IG%)]

The weighting coefficients used in the HIGS formula were prespecified according to the previously described HIGS framework and were not re-estimated or optimized within the present cohort. This approach was preferred to maintain methodological consistency with the original score definition and to reduce the risk of data-driven overfitting in a relatively small retrospective cohort. The use of unequal weights was based on the different biological and prognostic contributions of the three components. RDW was assigned the highest weight because it reflects multiple pathophysiological processes, including systemic inflammation, oxidative stress, tissue hypoxia, and erythrocyte heterogeneity, all of which have been associated with adverse cardiovascular outcomes. PDW was assigned an intermediate weight because it reflects platelet activation and thrombotic potential, whereas IG% was assigned a lower but complementary weight as a marker of acute inflammatory response and bone marrow activation. Therefore, HIGS should be regarded as a biologically guided composite index rather than a purely data-derived or mathematically optimized prediction score.

Receiver Operating Characteristic (ROC) curve analysis was performed to evaluate the prognostic performance of the HIGS score. The area under the curve (AUC) was calculated, and the optimal cut-off value was determined using the Youden index. Based on this analysis, sensitivity and specificity values were calculated to assess the discriminative ability of the model.

Logistic regression analyses were performed to identify factors associated with mortality and the need for PPI. Initially, all variables were evaluated using univariable logistic regression analysis. Variables found to be statistically significant in this analysis were subsequently included in the multivariable logistic regression model. For the PPI model, established TAVI-specific anatomical and procedural predictors, including membranous septum length, implantation depth, annular calcification grade, LVOT calcification grade, and valve oversizing, were included in the multivariable regression analysis because of their recognized clinical relevance for post-TAVI conduction disturbances. This approach was used to account for conventional anatomical and procedural risk factors and to evaluate whether HIGS was independently associated with PPI after adjustment for these established determinants. The results were reported as odds ratios (OR) with 95% confidence intervals (CI).

Model fit was evaluated using the Hosmer–Lemeshow goodness-of-fit test. In addition, the discriminative performance of the model was assessed using ROC curve analysis. The presence of multicollinearity in the multivariable model was evaluated using variance inflation factor (VIF) values, and VIF values were found to be low for all variables.

To assess whether the prognostic performance improved when the HIGS score was used together with conventional risk factors, a base model consisting of core clinical variables was first constructed, and the HIGS score was subsequently added to this model. The discriminative performance of these two models was compared using their respective AUC values.

To prevent exaggerated predictions due to overfitting, internal validation was performed. For this purpose, the bootstrap resampling method was used, and optimism-corrected AUC values were calculated for model performance. In addition, model calibration was evaluated using calibration plots, and the agreement between predicted and observed risks was analyzed.

Because the number of outcome events was limited, with 43 PPI events and 34 mortality events, the multivariable regression models were interpreted as exploratory risk-adjustment models rather than definitive predictive models. The potential risk of overfitting was considered during model construction, and internal validation using bootstrap resampling with optimism correction was performed to assess model stability. Nevertheless, the events-per-variable ratio was acknowledged as a methodological limitation, particularly in models including multiple clinical, procedural, anatomical, and hematological predictors.

#### Software

All statistical analyses were performed using Python (version 3.12). Data processing and cleaning were conducted using the pandas and numpy libraries, statistical analyses were performed with scipy and statsmodels, and ROC analysis and classification performance assessments were carried out using the scikit-learn package. The analysis workflow was conducted in accordance with principles of reproducibility.

## Results

During the study period, a total of 196 patients who underwent TAVI at our center and met the inclusion criteria were included in the analysis. The demographic characteristics, clinical comorbidities, and pre-procedural laboratory parameters of all patients were retrospectively evaluated. The baseline demographic, clinical, laboratory, electrocardiographic, echocardiographic, and procedural characteristics of the study population are presented in Tables 1–3.

**Table 1.** Baseline characteristics of the entire TAVI cohort (*n* = 196).

Variable	Value
**Demographic characteristics**	
Age (years)	78.16 ± 6.91
Male sex, n (%)	140 (71.4%)
Female sex, n (%)	56 (28.6%)
**Clinical characteristics**	
Diabetes mellitus, n (%)	100 (51.0%)
Hypertension, n (%)	169 (86.2%)
COPD, n (%)	64 (32.7%)
Atrial fibrillation, n (%)	11 (5.6%)
STS score	10.93 ± 6.15
HbA1c (%)	6.08 ± 0.66
**Electrocardiographic parameters**	
QRS duration (ms)	130.42 ± 13.93
PR interval (ms)	173.53 ± 32.22
Right bundle branch block, n (%)	10 (5.1%)
**Echocardiographic parameters**	
Ejection fraction (%)	46.03 ± 11.29
Aortic valve area (cm²)	0.85 ± 0.08
Peak gradient (mmHg)	77.57 ± 17.97
Mean gradient (mmHg)	52.54 ± 12.42
**Hematological and biochemical parameters**	
Hemoglobin (g/dL)	13.27 ± 2.18
White blood cell count (×10³/µL)	8.26 ± 2.26
Neutrophils (×10³/µL)	5.27 ± 2.20
Lymphocytes (×10³/µL)	1.64 ± 0.61
Platelets (×10³/µL)	235.16 ± 93.33
RDW (%)	15.41 ± 1.91
PDW (fL)	40.81 ± 17.38
Creatinine (mg/dL)	1.04 ± 0.53
Urea (mg/dL)	52.41 ± 26.59
Albumin (g/dL)	4.09 ± 0.43
C-reactive protein (mg/L)	2.98 ± 5.46
**Procedural characteristics**	
Device diameter (mm)	28.39 ± 2.86
Predilatation, n (%)	137 (69.9%)
Postdilatation, n (%)	55 (28.1%)
**Clinical outcomes**	
Permanent pacemaker implantation, n (%)	43 (21.9%)
Mortality, n (%)	34 (17.3%)

Descriptive statistical methods were used to summarize the baseline characteristics of the study population. Continuous variables are presented as mean ± standard deviation (SD), whereas categorical variables are expressed as number (n) and percentage (%). Since Table 1 summarizes the overall characteristics of the entire cohort without group comparisons, no comparative statistical tests were applied. In tables where comparative analyses are performed, statistical tests may include the Student’s t-test or Mann–Whitney U test for continuous variables and the Chi-square or Fisher’s exact test for categorical variables. In such analyses, statistically significant values are indicated in bold font. The p-value represents the probability that the observed difference occurred by chance, and values <0.05 are considered statistically significant. STS = Society of Thoracic Surgeons risk score, COPD = Chronic Obstructive Pulmonary Disease, HbA1c = Glycated Hemoglobin, QRS = QRS complex duration on electrocardiography, PR = PR interval on electrocardiography, RDW = Red Cell Distribution Width, PDW = Platelet Distribution Width, CRP = C-Reactive Protein, EF = Ejection Fraction, TAVI = Transcatheter Aortic Valve Implantation.

**Table 2.** Comparison of baseline variables according to outcomes: PPI and mortality (*n* = 196).

Variable	No PPI (*n* = 153)	PPI (*n* = 43)	*p*	Alive (*n* = 162)	Ex (*n* = 34)	*p*
Age (years)	78.12 ± 6.95	78.30 ± 6.79	0.88	77.58 ± 6.71	80.76 ± 7.42	**0.018**
Male sex, n (%)	111 (72.5%)	29 (67.4%)	0.48	115 (71.0%)	25 (73.5%)	0.77
Female, n (%)	42 (27.5%)	14 (32.6%		47 (29.0%)	9 (26.5%)	
STS score	10.41 ± 5.98	12.67 ± 6.41	**0.041**	10.01 ± 5.64	15.09 ± 7.13	**<0.001**
QRS duration (ms)	128.63 ± 12.11	136.70 ± 17.83	**0.003**	129.11 ± 12.91	136.46 ± 17.28	**0.012**
PR interval (ms)	170.66 ± 30.42	183.78 ± 36.17	**0.021**	171.42 ± 31.11	183.76 ± 35.84	**0.034**
Right bundle branch block, n (%)	5 (3.3%)	5 (11.6%)	**0.028**	6 (3.7%)	4 (11.8%)	**0.047**
Atrial fibrillation, n (%)	7 (4.6%)	4 (9.3%)	0.22	7 (4.3%)	4 (11.8%)	**0.049**
Ejection fraction (%)	46.89 ± 11.14	43.12 ± 11.49	0.062	47.12 ± 10.92	41.11 ± 11.88	**0.007**
Aortic valve area (cm²)	0.85 ± 0.08	0.84 ± 0.07	0.39	0.86 ± 0.08	0.82 ± 0.07	**0.013**
Peak gradient (mmHg)	77.81 ± 17.36	76.67 ± 20.01	0.73	78.40 ± 17.08	73.82 ± 21.54	0.21
Mean gradient (mmHg)	52.87 ± 12.20	51.35 ± 13.30	0.46	53.41 ± 11.90	48.76 ± 14.02	**0.041**
HbA1c (%)	6.04 ± 0.64	6.20 ± 0.71	0.17	6.02 ± 0.62	6.34 ± 0.76	**0.018**
Creatinine (mg/dL)	1.00 ± 0.48	1.20 ± 0.64	**0.028**	0.97 ± 0.42	1.39 ± 0.71	**<0.001**
Urea (mg/dL)	49.98 ± 24.78	61.18 ± 30.83	**0.019**	48.21 ± 23.44	72.00 ± 33.12	**<0.001**
Albumin (g/dL)	4.13 ± 0.42	3.94 ± 0.46	**0.015**	4.16 ± 0.41	3.77 ± 0.48	**<0.001**
RDW (%)	15.22 ± 1.80	16.07 ± 2.13	**0.014**	15.11 ± 1.71	16.82 ± 2.21	**<0.001**
PDW (fL)	39.12 ± 16.01	46.81 ± 20.62	**0.018**	38.94 ± 15.44	48.84 ± 22.61	**0.003**
IG%	0.62 ± 0.27	0.81 ± 0.32	**0.002**	0.61 ± 0.25	0.88 ± 0.34	**<0.001**
HIGS score	−0.08 ± 0.52	0.63 ± 0.71	**<0.001**	−0.12 ± 0.48	0.84 ± 0.76	**<0.001**

Statistical analyses were performed to compare baseline variables according to permanent pacemaker implantation (PPI) and mortality outcomes. Continuous variables are presented as mean ± standard deviation (SD) and were compared using the Student’s t-test for normally distributed variables or the Mann–Whitney U test when appropriate. Categorical variables are expressed as number (n) and percentage (%) and were analyzed using the Chi-square test or Fisher’s exact test where applicable. In the table, statistically significant values are indicated in bold font. The p-value represents the probability that the observed difference occurred by chance, and values less than 0.05 are considered statistically significant. PPI = Permanent Pacemaker Implantation, STS = Society of Thoracic Surgeons risk score, QRS = QRS complex duration on electrocardiography, PR = PR interval on electrocardiography, RBBB = Right Bundle Branch Block, EF = Ejection Fraction, HbA1c = Glycated Hemoglobin, RDW = Red Cell Distribution Width, PDW = Platelet Distribution Width, IG = Immature Granulocyte percentage, HIGS = Hematological Inflammatory Gradient Score.

**Table 3.** Procedural and valve-related characteristics according to outcomes: PPI and mortality (*n* = 196).

Variable	No PPI (*n* = 153)	PPI (*n* = 43)	*p*	Alive (*n* = 162)	Ex (*n* = 34)	*p*
**Device type**			**0.041**			0.33
Acurate Neo, n (%)	52 (34.0%)	9 (20.9%)		51 (31.5%)	10 (29.4%)	
Evolut R, n (%)	64 (41.8%)	25 (58.1%)		72 (44.4%)	17 (50.0%)	
Portico, n (%)	23 (15.0%)	6 (14.0%)		25 (15.4%)	4 (11.8%)	
Myval THV, n (%)	14 (9.2%)	3 (7.0%)		14 (8.7%)	3 (8.8%)	
Device diameter (mm)	28.11 ± 2.72	29.38 ± 3.12	**0.015**	28.21 ± 2.75	29.21 ± 3.18	0.09
Predilatation, n (%)	103 (67.3%)	34 (79.1%)	0.13	114 (70.4%)	23 (67.6%)	0.74
Postdilatation, n (%)	35 (22.9%)	20 (46.5%)	**0.002**	42 (25.9%)	13 (38.2%)	0.12
Membranous septum length (mm)	7.63 ± 1.15	6.12 ± 1.11	**<0.001**	7.26 ± 1.30	7.50 ± 1.30	0.334
Implantation depth (mm)	4.71 ± 1.18	6.95 ± 1.27	**<0.001**	5.24 ± 1.48	5.05 ± 1.69	0.484
Annular calcification grade (0–3)	0.94 ± 0.90	1.19 ± 0.76	**0.039**	0.93 ± 0.86	1.32 ± 0.88	0.017
LVOT calcification grade (0–3)	0.61 ± 0.70	1.42 ± 0.98	**<0.001**	0.75 ± 0.84	1.00 ± 0.82	0.062
Valve oversizing (%)	8.62 ± 2.52	10.60 ± 2.22	**<0.001**	9.02 ± 2.65	9.21 ± 2.23	0.664

Comparative statistical analyses were performed to evaluate procedural, valve-related, and TAVI-specific anatomical characteristics according to permanent pacemaker implantation (PPI) and mortality outcomes. Continuous variables are presented as mean ± standard deviation (SD). Normally distributed continuous variables were compared using the independent-samples t-test, whereas non-normally distributed or ordinal variables were compared using the Mann–Whitney U test. Categorical variables are expressed as number (n) and percentage (%) and were analyzed using the Chi-square test or Fisher’s exact test, as appropriate. Statistically significant p-values are indicated in bold font. PPI = Permanent Pacemaker Implantation, THV = Transcatheter Heart Valve, LVOT = Left Ventricular Outflow Tract.

Patients were grouped according to permanent pacemaker implantation status and in-hospital mortality, and comparisons between groups were performed.

Table 1 presents the baseline characteristics of the entire TAVI cohort consisting of 196 patients. The mean age of the study population was 78.16 ± 6.91 years. The majority of patients were male, accounting for 71.4% of the cohort, while females represented 28.6%. Diabetes mellitus was present in 51.0% of patients, hypertension in 86.2%, COPD in 32.7%, and atrial fibrillation in 5.6%. The mean STS score was 10.93 ± 6.15. Electrocardiographic parameters showed a mean QRS duration of 130.42 ± 13.93 ms and a mean PR interval of 173.53 ± 32.22 ms, while right bundle branch block was observed in 5.1% of patients. Echocardiographic evaluation revealed a mean left ventricular ejection fraction of 46.03 ± 11.29%. The mean aortic valve area was 0.85 ± 0.08 cm^2^, with a peak gradient of 77.57 ± 17.97 mmHg and a mean gradient of 52.54 ± 12.42 mmHg. Laboratory findings demonstrated a mean hemoglobin level of 13.27 ± 2.18 g/dL, HbA1c level of 6.08 ± 0.66%, white blood cell count of 8.26 ± 2.26 × 10³/µL, white blood cell count of 8.26 ± 2.26 × 10³/µL, neutrophil count of 5.27 ± 2.20 × 10³/µL, lymphocyte count of 1.64 ± 0.61 × 10³/µL, and platelet count of 235.16 ± 93.33 × 10³/µL. The mean RDW was 15.41 ± 1.91%, PDW was 40.81 ± 17.38 fL, creatinine was 1.04 ± 0.53 mg/dL, urea was 52.41 ± 26.59 mg/dL, albumin was 4.09 ± 0.43 g/dL, and C-reactive protein was 2.98 ± 5.46 mg/L. Regarding procedural characteristics, the mean device diameter was 28.39 ± 2.86 mm. Predilatation was performed in 69.9% of patients, whereas postdilatation was performed in 28.1%. In terms of clinical outcomes, permanent pacemaker implantation occurred in 21.9% of patients and overall mortality was observed in 17.3% of the cohort (Table 1).

According to Table 2, patients with adverse outcomes demonstrated significantly higher STS scores, prolonged QRS duration and PR interval, and a higher prevalence of right bundle branch block. The STS score was significantly higher in both the PPI group and the mortality group (12.67 ± 6.41 vs 10.41 ± 5.98, *p* = 0.041; and 15.09 ± 7.13 vs 10.01 ± 5.64, *p* < 0.001). QRS duration was significantly longer in patients with PPI and mortality (136.70 ± 17.83 vs 128.63 ± 12.11 ms, *p* = 0.003; and 136.46 ± 17.28 vs 129.11 ± 12.91 ms, *p* = 0.012). PR interval was also significantly prolonged in the PPI and mortality groups (183.78 ± 36.17 vs 170.66 ± 30.42 ms, *p* = 0.021; and 183.76 ± 35.84 vs 171.42 ± 31.11 ms, *p* = 0.034). Mortality was additionally associated with older age (80.76 ± 7.42 vs 77.58 ± 6.71 years, *p* = 0.018), atrial fibrillation (11.8% vs 4.3%, *p* = 0.049), lower ejection fraction (41.11 ± 11.88% vs 47.12 ± 10.92%, *p* = 0.007), smaller aortic valve area (0.82 ± 0.07 vs 0.86 ± 0.08 cm^2^, *p* = 0.013), and lower mean transvalvular gradient (48.76 ± 14.02 vs 53.41 ± 11.90 mmHg, *p* = 0.041). Creatinine and urea levels were significantly elevated in both the PPI and mortality groups, whereas albumin levels were significantly lower. Inflammatory and hematological parameters, including RDW, PDW, IG%, and HIGS score, were significantly higher in patients who developed PPI or experienced mortality (Table 2).

**Figure 1.**
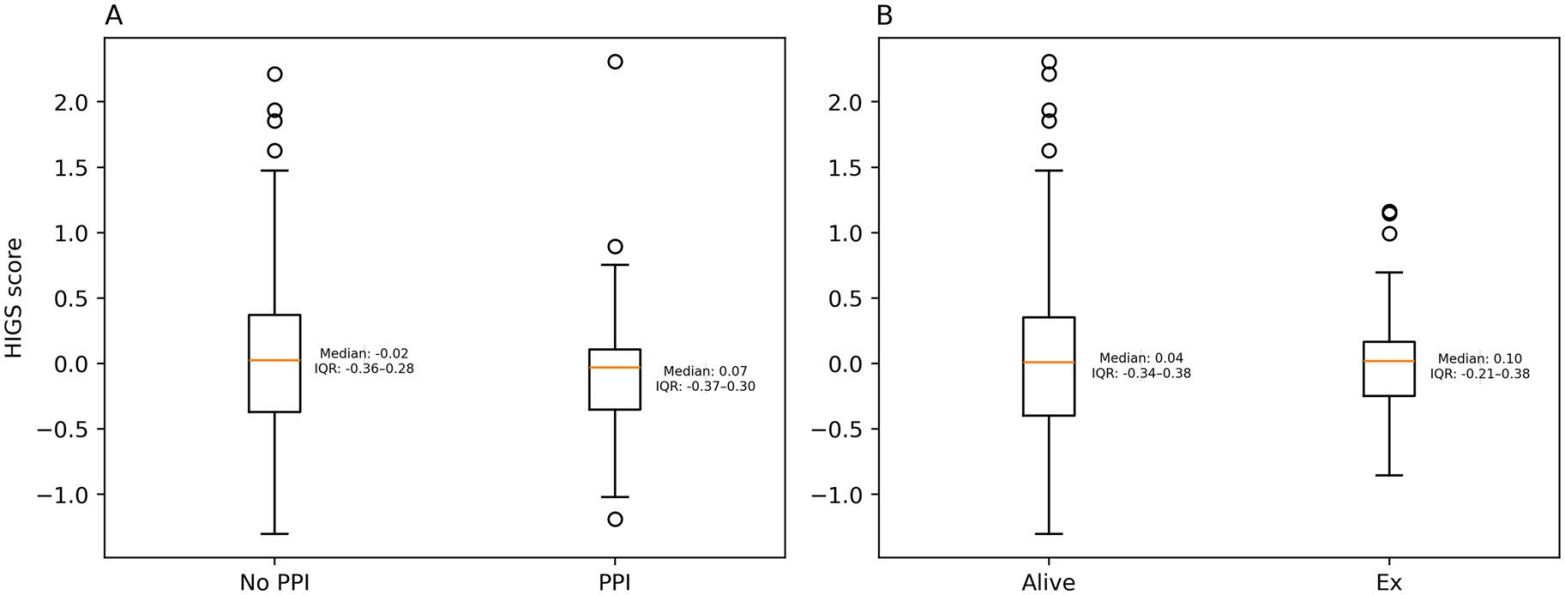
Distribution of HIGS scores according to clinical outcomes after TAVI. (A) Comparison of Hematological Inflammatory Gradient Score (HIGS) values between patients with and without permanent pacemaker implantation (PPI). (B) Comparison of HIGS values between survivors (Alive) and patients who died during follow-up (Ex). Box plots represent the median (central line), interquartile range (box), and range (whiskers), while individual circles indicate outliers. Higher HIGS values were observed in patients who developed PPI and in those who died compared with their respective counterparts. HIGS = Hematological Inflammatory Gradient Score; PPI = Permanent Pacemaker Implantation.

As shown in Figure 1, HIGS scores differed according to clinical outcomes. Patients who developed PPI demonstrated higher HIGS values compared with those without PPI. Similarly, patients who died exhibited higher HIGS scores compared with survivors. The distribution of HIGS values demonstrated generally higher median values among patients with adverse outcomes, including both PPI and mortality (Figure 1).

As shown in Table 3, device type distribution differed significantly between patients with and without PPI (*p* = 0.041). The use of the Evolut R valve was more frequent in the PPI group compared with the No PPI group (58.1% vs 41.8%), whereas the Acurate Neo valve was more commonly used in the No PPI group (34.0% vs 20.9%). Device diameter was significantly larger in patients who developed PPI compared with those who did not (29.38 ± 3.12 mm vs 28.11 ± 2.72 mm, *p* = 0.015). Postdilatation was also significantly more frequent in the PPI group than in the No PPI group (46.5% vs 22.9%, *p* = 0.002).

TAVI-specific anatomical and procedural predictors also differed significantly according to PPI status. Patients who required PPI had a significantly shorter membranous septum length compared with those without PPI (6.12 ± 1.11 mm vs 7.63 ± 1.15 mm, *p* < 0.001), greater implantation depth (6.95 ± 1.27 mm vs 4.71 ± 1.18 mm, *p* < 0.001), higher annular calcification grade (1.19 ± 0.76 vs 0.94 ± 0.90, *p* = 0.039), higher LVOT calcification grade (1.42 ± 0.98 vs 0.61 ± 0.70, *p* < 0.001), and greater valve oversizing (10.60 ± 2.22% vs 8.62 ± 2.52%, *p* < 0.001). In the mortality analysis, most procedural and anatomical variables were comparable between survivors and non-survivors; however, annular calcification grade was significantly higher in patients who died compared with survivors (1.32 ± 0.88 vs 0.93 ± 0.86, *p* = 0.017). LVOT calcification grade showed a numerically higher value in the mortality group, although this difference did not reach statistical significance (1.00 ± 0.82 vs 0.75 ± 0.84, *p* = 0.062) (Table 3).

The univariable logistic regression analysis demonstrated that STS score, QRS duration, PR interval, right bundle branch block, creatinine, urea, albumin, RDW, PDW, IG%, HIGS score, device diameter, postdilatation, membranous septum length, implantation depth, LVOT calcification grade, and valve oversizing were significantly associated with PPI. Specifically, higher STS score (OR: 1.06, 95% CI: 1.01–1.11, *p* = 0.024), prolonged QRS duration (OR: 1.04, 95% CI: 1.01–1.06, *p* = 0.002), prolonged PR interval (OR: 1.02, 95% CI: 1.01–1.03, *p* = 0.006), presence of right bundle branch block (OR: 3.87, 95% CI: 1.09–13.72, *p* = 0.036), elevated creatinine (OR: 1.63, 95% CI: 1.05–2.52, *p* = 0.028), increased urea levels (OR: 1.02, 95% CI: 1.01–1.03, *p* = 0.015), lower albumin levels (OR: 0.49, 95% CI: 0.27–0.90, *p* = 0.022), higher RDW (OR: 1.23, 95% CI: 1.06–1.42, *p* = 0.006), higher PDW (OR: 1.03, 95% CI: 1.01–1.06, *p* = 0.014), higher IG percentage (OR: 6.41, 95% CI: 2.35–17.45, *p* < 0.001), higher HIGS score (OR: 5.72, 95% CI: 2.89–11.31, *p* < 0.001), larger device diameter (OR: 1.17, 95% CI: 1.03–1.33, *p* = 0.015), and the use of postdilatation (OR: 2.92, 95% CI: 1.45–5.88, *p* = 0.003) were associated with an increased likelihood of PPI.

Among TAVI-specific anatomical and procedural predictors, shorter membranous septum length was associated with a lower likelihood of PPI per millimeter increase, indicating a protective association with longer septal length (OR: 0.31, 95% CI: 0.21–0.46, *p* < 0.001). In contrast, greater implantation depth (OR: 4.73, 95% CI: 2.92–7.66, *p* < 0.001), higher LVOT calcification grade (OR: 3.12, 95% CI: 2.00–4.87, *p* < 0.001), and greater valve oversizing (OR: 1.40, 95% CI: 1.20–1.64, *p* < 0.001) were significantly associated with an increased likelihood of PPI. Annular calcification grade was not significantly associated with PPI in univariable analysis (OR: 1.37, 95% CI: 0.94–2.00, *p* = 0.106).

For mortality, age, STS score, QRS duration, PR interval, right bundle branch block, atrial fibrillation, lower ejection fraction, smaller aortic valve area, lower mean gradient, higher creatinine, higher urea, lower albumin, higher HbA1c, higher RDW, higher PDW, higher IG percentage, higher HIGS score, and higher annular calcification grade were significantly associated with mortality. Among the newly incorporated TAVI-specific anatomical variables, annular calcification grade was significantly associated with mortality (OR: 1.65, 95% CI: 1.09–2.51, *p* = 0.018), whereas membranous septum length, implantation depth, LVOT calcification grade, and valve oversizing were not significantly associated with mortality. The strongest inflammatory and hematological associations for mortality were observed for IG percentage (OR: 7.84, 95% CI: 3.12–19.67, *p* < 0.001) and HIGS score (OR: 7.36, 95% CI: 3.61–15.01, *p* < 0.001) (Table 4).

**Table 4.** Univariable logistic regression analyses for predicting PPI and mortality.

	Panel A: PPI			Panel B: Mortality		
Variable	OR	95% CI	*p*	OR	95% CI	*p*
Age (years)	1.01	0.97 − 1.05	0.63	1.07	1.02 − 1.13	**0.008**
STS score	1.06	1.01 − 1.11	**0.024**	1.12	1.06 − 1.19	**<0.001**
QRS duration	1.04	1.01 − 1.06	**0.002**	1.03	1.01 − 1.05	**0.011**
PR interval	1.02	1.01 − 1.03	**0.006**	1.01	1.00 − 1.02	**0.031**
RBBB	3.87	1.09 − 13.72	**0.036**	3.52	1.02 − 12.13	**0.046**
AF	2.12	0.64 − 7.03	0.21	2.97	1.03 − 8.55	**0.044**
EF	0.97	0.94 − 1.01	0.10	0.94	0.91 − 0.98	**0.003**
AVA	0.74	0.34 − 1.61	0.45	0.39	0.18 − 0.86	**0.020**
Mean gradient	0.99	0.97 − 1.01	0.28	0.97	0.95 − 0.99	**0.018**
Creatinine	1.63	1.05 − 2.52	**0.028**	2.41	1.55 − 3.73	**<0.001**
Urea	1.02	1.01 − 1.03	**0.015**	1.04	1.02 − 1.06	**<0.001**
Albumin	0.49	0.27 − 0.90	**0.022**	0.30	0.17 − 0.55	**<0.001**
HbA1c	1.33	0.92 − 1.92	0.13	1.71	1.14 − 2.56	**0.009**
RDW	1.23	1.06 − 1.42	**0.006**	1.49	1.27 − 1.75	**<0.001**
PDW	1.03	1.01 − 1.06	**0.014**	1.05	1.02 − 1.08	**0.002**
IG%	6.41	2.35 − 17.45	**<0.001**	7.84	3.12 − 19.67	**<0.001**
HIGS	5.72	2.89 − 11.31	**<0.001**	7.36	3.61 − 15.01	**<0.001**
Device type	1.18	0.87 − 1.60	0.29	1.09	0.79 − 1.49	0.59
Device diameter	1.17	1.03 − 1.33	**0.015**	1.11	0.96 − 1.28	0.15
Predilatation	1.78	0.83 − 3.83	0.14	0.88	0.41 − 1.91	0.74
Postdilatation	2.92	1.45 − 5.88	**0.003**	1.77	0.81 − 3.85	0.15
Membranous septum length (mm)	0.31	0.21–0.46	**<0.001**	1.16	0.86–1.54	0.328
Implantation depth (mm)	4.73	2.92–7.66	**<0.001**	0.92	0.72–1.18	0.519
Annular calcification grade (0–3)	1.37	0.94–2.00	**0.106**	1.65	1.09–2.51	0.018
LVOT calcification grade (0–3)	3.12	2.00–4.87	**<0.001**	1.41	0.92–2.14	0.111
Valve oversizing (%)	1.40	1.20–1.64	**<0.001**	1.03	0.89–1.19	0.696

Univariable logistic regression analyses were performed to identify variables associated with permanent pacemaker implantation (PPI) and mortality. Results are presented as odds ratios (ORs) with corresponding 95% confidence intervals (CIs). Continuous variables were analyzed as numerical predictors within the regression model, whereas categorical variables were entered as binary variables. Ordinal anatomical variables, including annular calcification grade and LVOT calcification grade, were analyzed as ordinal numerical predictors. The p-value represents the statistical significance of the association between each predictor and the outcome, and values less than 0.05 were considered statistically significant. In the table, statistically significant results are indicated in bold font. OR values greater than 1 indicate an increased likelihood of the outcome, whereas OR values less than 1 indicate a protective or inverse association. OR = Odds Ratio, CI = Confidence Interval, PPI = Permanent Pacemaker Implantation, STS = Society of Thoracic Surgeons risk score, QRS = QRS complex duration on electrocardiography, PR = PR interval on electrocardiography, RBBB = Right Bundle Branch Block, AF = Atrial Fibrillation, EF = Ejection Fraction, AVA = Aortic Valve Area, RDW = Red Cell Distribution Width, PDW = Platelet Distribution Width, IG = Immature Granulocyte percentage, HIGS = Hematological Inflammatory Global Score, LVOT = Left Ventricular Outflow Tract.

The multivariable logistic regression analysis demonstrated that, after adjustment for clinical, electrocardiographic, procedural, and TAVI-specific anatomical predictors, membranous septum length, implantation depth, LVOT calcification grade, valve oversizing, and HIGS score remained independently associated with PPI. Longer membranous septum length was independently associated with a lower likelihood of PPI (OR: 0.37, 95% CI: 0.18–0.73, *p* = 0.004), whereas greater implantation depth (OR: 6.69, 95% CI: 3.07–14.56, *p* < 0.001), higher LVOT calcification grade (OR: 4.84, 95% CI: 1.91–12.25, *p* < 0.001), and greater valve oversizing (OR: 2.43, 95% CI: 1.57–3.77, *p* < 0.001) were independently associated with an increased likelihood of PPI. Importantly, HIGS score also remained independently associated with PPI in this adjusted model (OR: 3.96, 95% CI: 1.79–8.76, *p* < 0.001). QRS duration and Evolut valve use were associated with PPI in the initial adjusted model; however, after incorporation of detailed anatomical predictors, the strongest independent determinants were membranous septum length, implantation depth, LVOT calcification grade, valve oversizing, and HIGS score.

For mortality, STS score (OR: 1.08, 95% CI: 1.02–1.15, *p* = 0.010), creatinine level (OR: 1.74, 95% CI: 1.06–2.85, *p* = 0.028), lower albumin level (OR: 0.47, 95% CI: 0.24–0.92, *p* = 0.026), annular calcification grade (OR: 2.01, 95% CI: 1.19–3.39, *p* = 0.009), and HIGS score (OR: 4.92, 95% CI: 2.12–11.41, *p* < 0.001) were identified as independent predictors of mortality (Table 5).

**Table 5.** Multivariable logistic regression models for predicting PPI and mortality.

	OR	95% CI	*p*	OR	95% CI	*p*
Variable	Panel A: PPI Model			Panel B: Mortality Model		
Age	1.01	0.97–1.05	0.64	1.05	0.99–1.11	0.09
QRS duration	1.02	1.00–1.05	**0.041**	—	—	—
RBBB	2.36	0.61–9.15	0.21	—	—	—
Device type (Evolut vs others)	2.48	1.14–5.40	**0.022**	—	—	—
Device diameter	1.11	0.98–1.26	0.10	—	—	—
Postdilatation	2.01	0.94–4.29	0.07	—	—	—
**Membranous septum length (mm)**	**0.37**	**0.18–0.73**	**0.004**	—	—	—
**Implantation depth (mm)**	**6.69**	**3.07–14.56**	**<0.001**	—	—	—
**Annular calcification grade (0–3)**	0.96	0.41–2.25	0.93	**2.01**	**1.19–3.39**	**0.009**
**LVOT calcification grade (0–3)**	**4.84**	**1.91–12.25**	**<0.001**	1.42	0.88–2.30	0.15
**Valve oversizing (%)**	**2.43**	**1.57–3.77**	**<0.001**	1.01	0.87–1.17	0.90
STS score	—	—	—	1.08	1.02–1.15	**0.010**
Creatinine	—	—	—	1.74	1.06–2.85	**0.028**
Albumin	—	—	—	0.47	0.24–0.92	**0.026**
HIGS	**3.96**	**1.79–8.76**	**<0.001**	**4.92**	**2.12–11.41**	**<0.001**

Multivariable logistic regression analyses were performed to identify independent predictors of permanent pacemaker implantation (PPI) and mortality. Variables that were statistically significant in univariable analysis or considered clinically relevant were entered into the multivariable models. In response to the recognized TAVI-specific determinants of conduction disturbances, membranous septum length, implantation depth, annular calcification grade, LVOT calcification grade, and valve oversizing were additionally incorporated into the adjusted PPI model. For the mortality model, clinically relevant baseline variables and anatomical predictors associated with mortality in univariable analysis were included. The results are presented as odds ratios (ORs) with corresponding 95% confidence intervals (CIs). Continuous variables were included as numerical predictors, whereas categorical variables were entered as binary variables. Ordinal anatomical variables, including annular calcification grade and LVOT calcification grade, were analyzed as ordinal numerical predictors. Statistically significant values are indicated in bold font. OR values greater than 1 indicate an increased likelihood of the outcome, whereas OR values less than 1 indicate a protective or inverse association. OR = Odds Ratio, CI = Confidence Interval, PPI = Permanent Pacemaker Implantation, STS = Society of Thoracic Surgeons risk score, QRS = QRS complex duration on electrocardiography, RBBB = Right Bundle Branch Block, HIGS = Hematological Inflammatory Global Score, LVOT = Left Ventricular Outflow Tract.

ROC analysis demonstrated that QRS duration (AUC: 0.69, 95% CI: 0.61–0.77, *p* = 0.002), PR interval (AUC: 0.65, 95% CI: 0.56–0.73, *p* = 0.009), STS score (AUC: 0.61, 95% CI: 0.52–0.70, *p* = 0.041), and device diameter (AUC: 0.63, 95% CI: 0.54–0.72, *p* = 0.028) were significant predictors of PPI. The HIGS score demonstrated the highest discriminative performance for predicting PPI with an AUC of 0.84 (95% CI: 0.77–0.91, *p* < 0.001), with an optimal cut-off value of >0.31, sensitivity of 81.4%, and specificity of 77.8%. For mortality prediction, STS score (AUC: 0.74, 95% CI: 0.66–0.82, *p* < 0.001), albumin (AUC: 0.76, 95% CI: 0.68–0.84, *p* < 0.001), RDW (AUC: 0.78, 95% CI: 0.71–0.86, *p* < 0.001), and IG percentage (AUC: 0.81, 95% CI: 0.74–0.88, *p* < 0.001) were significant predictors of mortality. The HIGS score demonstrated the highest predictive performance with an AUC of 0.87 (95% CI: 0.81–0.93, *p* < 0.001), with a cut-off value of >0.45, sensitivity of 85.3%, and specificity of 79.0% (Table 6).

**Table 6.** ROC analysis for prediction of PPI and mortality.

	AUC (95% CI)	Cut-off	Sensitivity (%)	Specificity (%)	*p*	AUC (95% CI)	Cut-off	Sensitivity (%)	Specificity (%)	*p*
Parameter	Panel A: PPI					Panel B: Mortality				
QRS duration	0.69 (0.61–0.77)	>133 ms	67.4	64.1	**0.002**	0.66 (0.57–0.75)	>134 ms	61.8	63.6	**0.011**
PR interval	0.65 (0.56–0.73)	>176 ms	60.5	62.7	**0.009**	0.63 (0.53–0.72)	>178 ms	58.8	60.5	**0.031**
STS score	0.61 (0.52–0.70)	>11.8	55.8	61.4	**0.041**	0.74 (0.66–0.82)	>13.2	73.5	69.1	**<0.001**
Device diameter	0.63 (0.54–0.72)	>29 mm	58.1	60.8	**0.028**	—	—	—	—	—
Albumin	—	—	—	—	—	0.76 (0.68–0.84)	<3.9 g/dL	76.5	71.6	**<0.001**
RDW	—	—	—	—	—	0.78 (0.71–0.86)	>16.1 %	79.4	72.8	**<0.001**
IG%	—	—	—	—	—	0.81 (0.74–0.88)	>0.74	82.4	74.7	**<0.001**
**HIGS score**	**0.84 (0.77–0.91)**	**>0.31**	**81.4**	**77.8**	**<0.001**	**0.87 (0.81–0.93)**	**>0.45**	**85.3**	**79.0**	**<0.001**

Receiver operating characteristic (ROC) curve analyses were performed to evaluate the predictive performance of selected variables for permanent pacemaker implantation (PPI) and mortality. The area under the ROC curve (AUC) with corresponding 95% confidence intervals (CI) was calculated to assess the discriminative ability of each parameter. Optimal cut-off values were determined using the Youden index, which maximizes the combined sensitivity and specificity. Sensitivity represents the proportion of true positive cases correctly identified by the model, whereas specificity represents the proportion of true negative cases correctly identified. In the table, statistically significant results are indicated in bold font, and p-values less than 0.05 were considered statistically significant. AUC = Area Under the Curve, CI = Confidence Interval, ROC = Receiver Operating Characteristic, PPI = Permanent Pacemaker Implantation, STS = Society of Thoracic Surgeons risk score, RDW = Red Cell Distribution Width, IG = Immature Granulocyte percentage, HIGS = Hematological Inflammatory Gradient Score.

**Figure 2.**
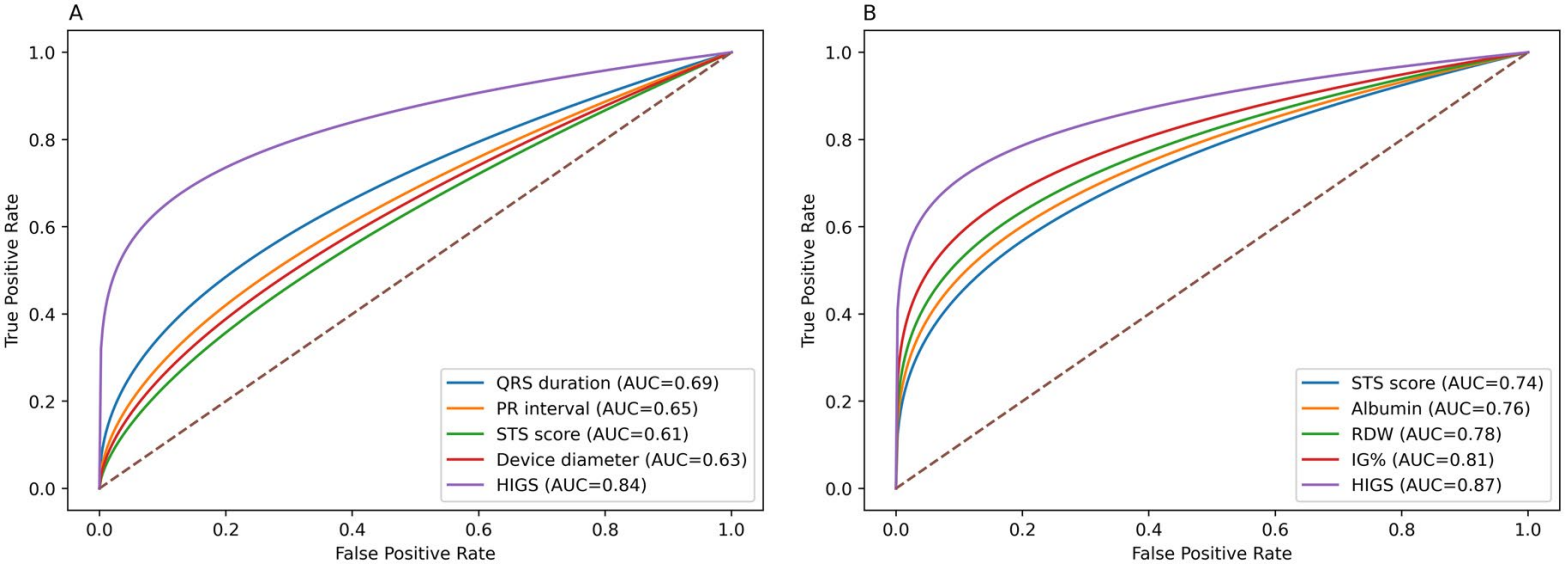
Receiver operating characteristic (ROC) curves for predicting permanent pacemaker implantation (PPI) and mortality after TAVI. (A) ROC curves comparing the predictive performance of QRS duration, PR interval, STS score, device diameter, and Hematological Inflammatory Gradient Score (HIGS) for PPI. HIGS demonstrated the highest discriminative ability with an AUC of 0.84 compared with QRS duration (AUC = 0.69), PR interval (AUC = 0.65), STS score (AUC = 0.61), and device diameter (AUC = 0.63). (B) ROC curves comparing predictors of mortality, including STS score, albumin, RDW, IG percentage, and HIGS. HIGS showed the best predictive performance with an AUC of 0.87, followed by IG% (AUC = 0.81), RDW (AUC = 0.78), albumin (AUC = 0.76), and STS score (AUC = 0.74). The diagonal dashed line represents the reference line indicating no discriminative ability (AUC = 0.50). AUC = Area Under the Curve; ROC = Receiver Operating Characteristic; STS = Society of Thoracic Surgeons risk score; RDW = Red Cell Distribution Width; IG = Immature Granulocyte percentage; HIGS = Hematological Inflammatory Gradient Score; PPI = Permanent Pacemaker Implantation.

As shown in Figure 2A, ROC curve analysis demonstrated that HIGS had the highest discriminative ability for predicting PPI, with an AUC of 0.84, compared with QRS duration (AUC = 0.69), PR interval (AUC = 0.65), STS score (AUC = 0.61), and device diameter (AUC = 0.63). Similarly, Figure 2B illustrates the ROC analysis for mortality prediction. HIGS exhibited the highest predictive performance with an AUC of 0.87, followed by IG percentage (AUC = 0.81), RDW (AUC = 0.78), albumin (AUC = 0.76), and STS score (AUC = 0.74) (Figure 2).

The incremental model analysis demonstrated that the addition of HIGS significantly improved the prediction of PPI. The base clinical/electrocardiographic/procedural model yielded an AUC of 0.72 (95% CI: 0.64–0.80), which increased to 0.85 (95% CI: 0.78–0.91) after the inclusion of HIGS (ΔAUC = 0.13, *p* = 0.002). Model calibration remained adequate, with Hosmer–Lemeshow p-values of 0.44 for the base model and 0.49 for the model including HIGS. The Brier score improved from 0.158 to 0.136 following the addition of HIGS. Similarly, for mortality prediction, the base clinical model demonstrated an AUC of 0.77 (95% CI: 0.69–0.85), which increased to 0.89 (95% CI: 0.83–0.94) after incorporating HIGS (ΔAUC = 0.12, *p* < 0.001). Model calibration remained acceptable with Hosmer–Lemeshow p-values of 0.39 and 0.46 for the base and extended models, respectively. The Brier score also improved from 0.145 to 0.118 after the addition of HIGS (Table 7).

**Table 7.** Incremental value of HIGS over conventional models for predicting PPI and mortality.

Model	AUC (95% CI)	ΔAUC	*p*	Hosmer–Lemeshow p	Brier Score
**Panel A: Prediction of PPI**					
Base clinical/electrocardiographic/procedural model	0.72 (0.64–0.80)	—	—	0.44	0.158
Base model + HIGS	**0.85 (0.78–0.91)**	**0.13**	**0.002**	0.49	0.136
**Panel B: Prediction of Mortality**					
Base clinical model	0.77 (0.69–0.85)	—	—	0.39	0.145
Base model + HIGS	**0.89 (0.83–0.94)**	**0.12**	**<0.001**	0.46	0.118

Incremental predictive performance analyses were performed to evaluate the additional value of the Hematological Inflammatory Gradient Score (HIGS) beyond conventional clinical and procedural models for predicting permanent pacemaker implantation (PPI) and mortality. Model discrimination was assessed using the area under the receiver operating characteristic curve (AUC) with corresponding 95% confidence intervals (CI). The change in AUC (ΔAUC) represents the improvement in discriminative ability after the addition of HIGS to the base model. Statistical significance of the improvement was evaluated using the DeLong test for comparison of correlated ROC curves. Model calibration was assessed using the Hosmer–Lemeshow goodness-of-fit test, where p-values greater than 0.05 indicate good model calibration. Overall model performance was additionally evaluated using the Brier score, which measures the accuracy of probabilistic predictions, with lower values indicating better predictive performance. In the table, statistically significant results are indicated in bold font, and p-values less than 0.05 were considered statistically significant. AUC = Area Under the Curve, CI = Confidence Interval, ΔAUC = Change in Area Under the Curve, PPI = Permanent Pacemaker Implantation, HIGS = Hematological Inflammatory Gradient Score.

**Figure 3.**
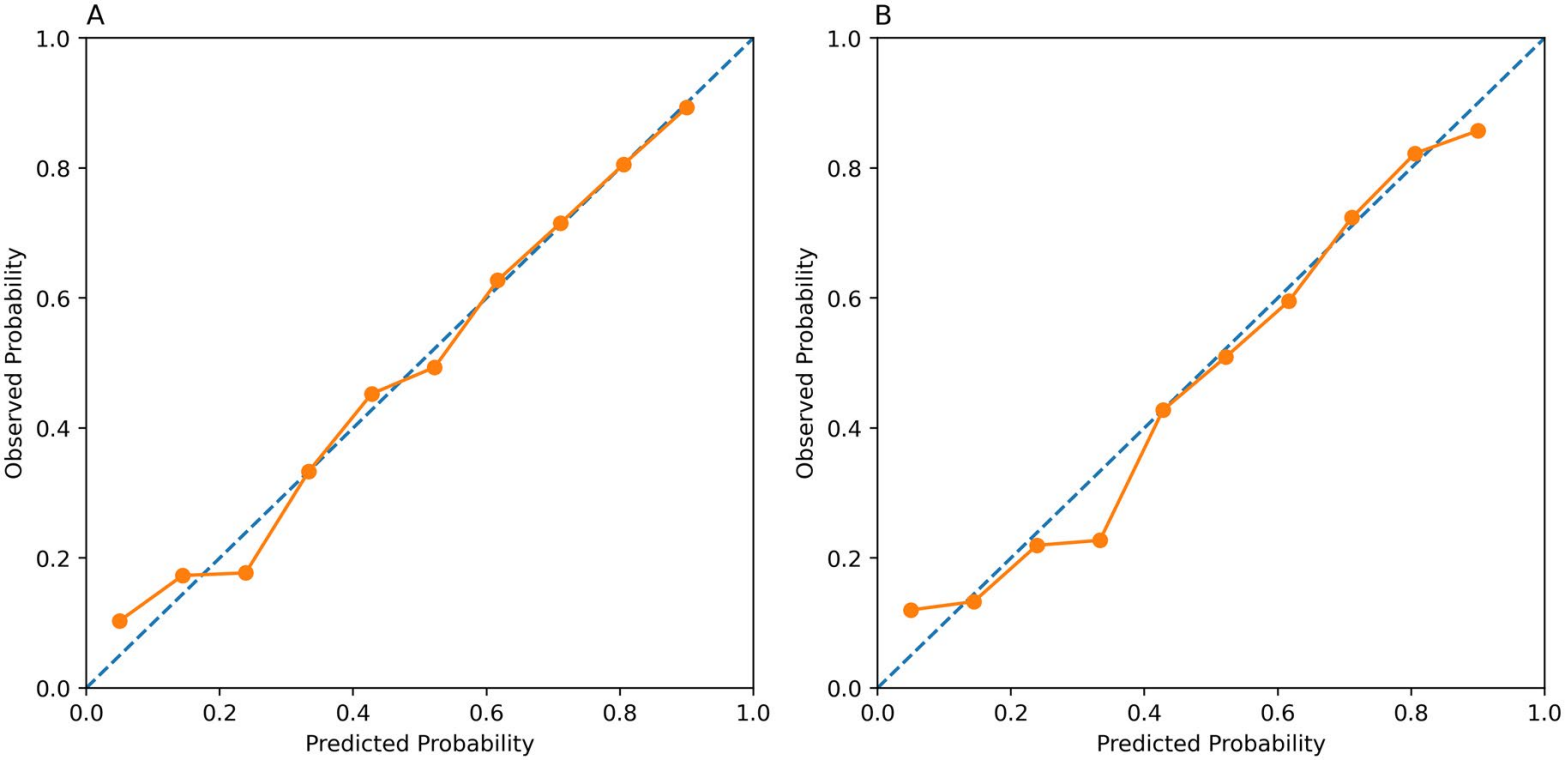
Calibration plots of the prediction models for permanent pacemaker implantation (PPI) and mortality after TAVI. (A) Calibration curve for the PPI prediction model. (B) Calibration curve for the mortality prediction model. The dashed diagonal line represents the ideal calibration where predicted probabilities perfectly match observed event rates. The solid line with points represents the observed calibration of the models across risk deciles. Both models demonstrate good agreement between predicted and observed probabilities, indicating appropriate model calibration.

Figure 3 presents the calibration plots of the prediction models for PPI and mortality. As shown in Figure 3A, the predicted probabilities for PPI closely correspond to the observed event rates across risk deciles, indicating good calibration of the model. Similarly, Figure 3B demonstrates a strong agreement between predicted and observed mortality probabilities, with calibration points lying close to the reference diagonal line (Figure 3).

**Figure 4.**
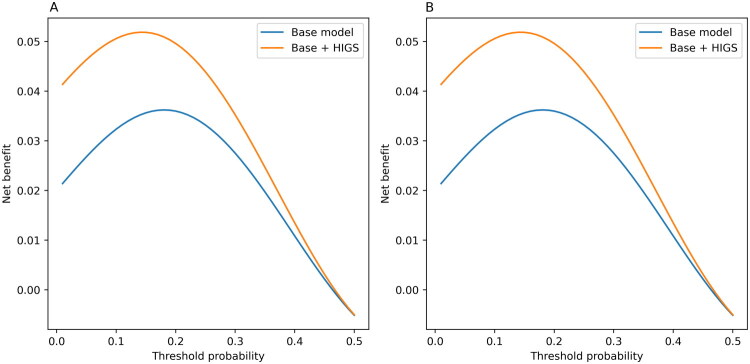
Decision curve analysis evaluating the clinical utility of HIGS for predicting permanent pacemaker implantation (PPI) and mortality after TAVI. (A) Decision curve analysis for the prediction of PPI. (B) Decision curve analysis for the prediction of mortality. The curves represent the net clinical benefit across a range of threshold probabilities for the base clinical models and the extended models including the Hematological Inflammatory Gradient Score (HIGS). The addition of HIGS to the base models provides a higher net benefit across a broad range of threshold probabilities compared with the base models alone, indicating improved clinical utility.

Figure 4 illustrates the decision curve analysis evaluating the clinical utility of the prediction models. As shown in Figure 4A, the model including HIGS demonstrated a higher net benefit across a wide range of threshold probabilities compared with the base model for predicting PPI. Similarly, Figure 4B shows that the addition of HIGS improved the net clinical benefit for mortality prediction compared with the base clinical model alone (Figure 4).

## Discussion

In this study, the prognostic value of the Hematological Inflammatory Global Score (HIGS) for predicting the need for permanent pacemaker implantation and mortality in patients undergoing transcatheter aortic valve implantation was evaluated. The main findings of our study can be summarized as follows: (i) the HIGS score was significantly associated with the requirement for permanent pacemaker implantation after TAVI, (ii) the HIGS score also demonstrated a significant association with mortality, (iii) ROC analysis showed that the HIGS score exhibited high discriminative performance in predicting both PPI and mortality, and (iv) each component of HIGS, including RDW, PDW, and IG%, was significantly associated with clinical outcomes. In addition, when evaluated together with conventional clinical variables, the HIGS score appeared to be a potential biomarker that could provide incremental value for risk stratification. These findings suggest that the inflammatory and hematological response in TAVI patients may influence not only mortality but also conduction system complications and the requirement for permanent pacemaker implantation.

One notable finding of the present study was the relatively high in-hospital mortality rate observed in the cohort. One notable finding of the present study was the relatively high in-hospital mortality rate of 17.3%. This rate is higher than that generally reported in contemporary TAVI registries and randomized studies, particularly those including lower-risk or more selectively treated patient populations. The difference may primarily be explained by the high-risk profile of our real-world cohort. The patients included in the present study were elderly, had a substantial burden of comorbidities, and demonstrated a relatively high mean STS score, indicating increased baseline procedural and clinical risk. In addition, the single-center real-world nature of the cohort may have resulted in the inclusion of patients with more complex clinical characteristics who may not be fully comparable to patients enrolled in contemporary low-risk TAVI trials or registries. Therefore, the observed mortality rate should not be interpreted as representative of all contemporary TAVI populations but rather as reflecting the specific high-risk profile of the present cohort. Accordingly, the prognostic value of HIGS in this study should be interpreted within the context of this high-risk real-world TAVI population. Previous studies evaluating inflammatory biomarkers in TAVI populations have primarily focused on markers such as C-reactive protein, neutrophil-to-lymphocyte ratio, and platelet-based indices. However, these markers reflect only specific aspects of the inflammatory cascade [12]. In contrast, the HIGS score integrates parameters reflecting erythrocyte morphology, platelet activation, and acute inflammatory response, thereby providing a more comprehensive representation of systemic inflammatory activity. This multidimensional structure may explain the strong discriminative performance observed in our study. Although the principal value of HIGS lies in its composite multidimensional structure, evaluation of individual components may still provide mechanistic insight into the relative contribution of inflammation, platelet activation, and bone marrow response to adverse outcomes after TAVI. The weighting structure of HIGS should also be interpreted within this biological framework. The score was not intended to replace data-driven risk models but to provide a simple, reproducible, and biologically plausible composite marker derived from routinely available hematological parameters. Because the coefficients were not optimized specifically for the present TAVI cohort, the observed prognostic value of HIGS should be considered supportive of its potential clinical relevance rather than definitive evidence that the current weighting scheme is optimal for TAVI patients.

The requirement for permanent pacemaker implantation following TAVI is considered one of the most important procedure-specific complications and may have broader clinical implications beyond a simple conduction system disorder [6–8]. Patients who develop PPI have been reported to experience longer hospital stays, higher rates of rehospitalization, and potentially impaired long-term cardiac function [8]. Furthermore, right ventricular pacing–induced ventricular dyssynchrony may lead to deterioration in left ventricular function and an increased risk of heart failure in the long term [16]. Therefore, identifying risk markers that can predict the requirement for permanent pacemaker implantation after TAVI is of considerable importance in clinical practice. In our study, the significant association between the HIGS score and the need for permanent pacemaker implantation supports the hypothesis that inflammatory and hematological responses may influence not only mortality but also conduction system complications.

Multiple pathophysiological mechanisms contribute to the development of complications after TAVI. Particularly in elderly patients with multiple comorbidities, systemic inflammation, endothelial dysfunction, microvascular circulatory disturbances, and thrombotic processes represent key factors influencing clinical outcomes [6]. Increased inflammatory activity may affect not only cardiovascular events but also procedural complications. Mechanical stress during the TAVI procedure, local tissue injury during valve implantation, and hemodynamic changes may trigger a systemic inflammatory response [17]. These processes may contribute to the development of complications such as conduction system injury and myocardial stress [6–8].

In our study, the strong association between the HIGS score and mortality appears consistent with these pathophysiological mechanisms. The HIGS score is based on a combination of hematological parameters representing three distinct biological processes: erythrocyte heterogeneity (RDW), platelet activation (PDW), and acute inflammatory response (IG%). Each of these parameters reflects different mechanisms involved in the pathophysiology of cardiovascular diseases [11]. Increased RDW values are considered indicators of systemic inflammation, oxidative stress, and tissue hypoxia. The effects of inflammatory cytokines on erythropoiesis may increase erythrocyte size heterogeneity, leading to elevated RDW levels [13]. This condition may impair microvascular circulation, reduce organ perfusion, and ultimately contribute to worse clinical outcomes.

Similarly, PDW is an indicator of platelet activation and is closely associated with atherothrombotic processes [13]. Increased platelet activation may contribute to the development of thrombotic complications and microvascular obstruction, particularly after interventional procedures such as TAVI. IG%, on the other hand, reflects the proportion of immature granulocytes released from the bone marrow into the circulation and is considered an important marker of acute inflammatory response [14,15]. Interestingly, IG% alone demonstrated numerically stronger associations with both mortality and PPI compared with the composite HIGS score in univariable analyses. This finding may suggest that acute inflammatory activation and bone marrow response have a particularly important role in adverse post-TAVI outcomes. Nevertheless, because HIGS integrates multiple complementary biological pathways including inflammation, erythrocyte heterogeneity, and platelet activation, its clinical relevance may depend more on multidimensional risk representation rather than superiority over isolated biomarkers in statistical comparisons. During surgical or interventional stress, inflammatory responses may increase bone marrow activity, leading to elevated IG% levels. Therefore, the combined evaluation of RDW, PDW, and IG% allows simultaneous assessment of multidimensional biological processes such as inflammation, thrombosis, and hypoxia.

Another notable mechanism is the relationship between inflammatory response and conduction system injury. Local tissue trauma and mechanical stress occurring after TAVI may not only exert direct compression effects but may also trigger the release of inflammatory mediators [17]. Increased inflammatory activity may be associated with interstitial edema, microvascular circulatory disturbances, and impairment of electrical conduction within the conduction tissue. Inflammatory infiltration and tissue edema within the interventricular septum may increase electrical conduction heterogeneity and predispose patients to conduction block. These mechanisms may partially explain the association observed between elevated inflammatory markers and the development of post-procedural conduction abnormalities. In this context, elevated hematological parameters reflecting inflammation may represent a biological susceptibility to conduction system injury. The association between the HIGS score and the development of PPI observed in our study suggests that inflammation may play a role in the development of conduction system complications following TAVI. In addition to mechanical compression and anatomical proximity to the conduction system, inflammatory processes may further exacerbate conduction disturbances after TAVI. Procedural trauma and local tissue stress may trigger an inflammatory cascade within the interventricular septum, leading to interstitial edema, microvascular dysfunction, and transient impairment of electrical conduction pathways. These inflammatory responses may be associated with increased vulnerability of the atrioventricular conduction system to injury, particularly in elderly patients with pre-existing structural and microvascular alterations [7–9,17]. Therefore, elevated inflammatory markers prior to the procedure may reflect an underlying biological susceptibility to conduction system complications. The strong association observed between HIGS and PPI in our cohort supports the hypothesis that systemic inflammatory status may contribute to the development of post-procedural conduction abnormalities.

From this perspective, the HIGS score represents a novel approach by integrating three hematological parameters reflecting different pathophysiological processes into a single composite index. The high discriminative performance of the HIGS score in ROC analysis in our study suggests that the combined evaluation of these parameters may provide a stronger prognostic signal than individual biomarkers alone. However, these findings should be interpreted cautiously given the relatively limited event count in the present cohort. Although the ROC analyses demonstrated relatively high sensitivity and specificity values for HIGS in predicting both mortality and PPI, these findings should not be interpreted as definitive evidence of clinical utility. In addition, several regression analyses demonstrated relatively wide confidence intervals, indicating limited precision of some effect estimates. Therefore, the observed associations should be interpreted cautiously until validated in larger prospective multicenter cohorts. Although internal validation techniques were applied, the possibility of statistical instability and overfitting cannot be completely excluded, particularly in multivariable models incorporating multiple clinical and procedural predictors. This concern is particularly relevant given the limited number of PPI and mortality events and the resulting constraints related to the events-per-variable ratio. Indeed, biological processes such as inflammation, thrombosis, and hypoxia are known to be closely interconnected in the development of cardiovascular complications [11,13,17].

Various clinical and electrocardiographic parameters have been identified to predict mortality and the development of PPI in patients undergoing TAVI. Advanced age, reduced ejection fraction, renal dysfunction, and pre-existing conduction abnormalities are among the major risk factors [7–9]. However, most existing risk models are primarily based on demographic and clinical parameters and do not sufficiently incorporate inflammatory biomarkers. This limitation may lead to inadequate risk stratification, particularly in patient groups with significant inflammatory activity.

In our study, the HIGS score appeared to provide incremental prognostic value when evaluated together with conventional risk factors. Although statistical significance remained limited in the multivariable model, the high discriminative performance of the HIGS score observed in ROC analysis suggests that this parameter may still have clinical relevance. Importantly, the HIGS score can be easily calculated from routine complete blood count parameters, does not require additional costs, and can be rapidly applied in clinical practice, which represents a major advantage. From a clinical perspective, early identification of patients at increased risk for conduction disturbances may have important implications for procedural planning and post-procedural monitoring strategies. Patients with elevated HIGS scores before TAVI may benefit from closer rhythm monitoring, more cautious valve positioning strategies, and careful evaluation of implantation depth during the procedure [6–9,18–22]. Furthermore, identifying patients with a higher inflammatory burden prior to TAVI may help clinicians anticipate the potential need for permanent pacemaker implantation and optimize peri-procedural management. Thus, the integration of HIGS into pre-procedural assessment may contribute to a more individualized risk stratification approach in patients undergoing TAVI.

In addition, procedural factors, device characteristics, and TAVI-specific anatomical predictors were evaluated in the present study. Device type distribution differed significantly according to PPI status, and Evolut R valves were more frequently used in patients who developed PPI. More importantly, established anatomical and procedural determinants of post-TAVI conduction disturbances, including shorter membranous septum length, greater implantation depth, higher LVOT calcification grade, and greater valve oversizing, were significantly associated with PPI. These findings are consistent with previous studies emphasizing the importance of the spatial relationship between the prosthetic valve and the conduction system, as well as the mechanical effects of valve implantation on the membranous septum and LVOT region [6–9].

In the adjusted regression model including these TAVI-specific predictors, membranous septum length, implantation depth, LVOT calcification grade, valve oversizing, and HIGS remained independently associated with PPI. This finding suggests that the association between HIGS and PPI was not solely explained by conventional anatomical or procedural risk factors. Rather, HIGS may reflect an additional biological vulnerability related to systemic inflammation, hematological activation, and tissue susceptibility to conduction system injury. Nevertheless, this interpretation should remain cautious because the present study was retrospective and the anatomical measurements were not adjudicated by an independent imaging core laboratory.

The mechanical characteristics of the valve system used during TAVI also play an important role in the development of conduction system disturbances. Self-expanding valve systems are known to exert a more prolonged and wider radial force on the interventricular septum and membranous septum compared with balloon-expandable valves [18]. This may result in more pronounced mechanical compression on critical anatomical structures of the conduction system, including the His bundle and left bundle branch. Indeed, several clinical studies have reported higher rates of PPI with self-expanding valve systems compared with balloon-expandable valves [18,19]. In our study, Evolut R valves, which represent a self-expanding system, were also used more frequently in the PPI group. This finding is consistent with previously reported mechanisms related to mechanical compression and implantation depth described in the literature.

Procedural technical factors may also play an important role in conduction system injury. In particular, balloon valvuloplasty performed during pre-dilatation and post-dilatation procedures following valve implantation may impose additional mechanical stress on septal structures [20]. High-pressure balloon expansion during pre-dilatation may create tension within the aortic annulus and surrounding fibrous structures, potentially causing temporary or permanent injury to the conduction system [21]. Similarly, post-dilatation procedures performed to optimize valve positioning may exert additional compression on septal tissues, thereby increasing the risk of PPI [22]. In our study, the incidence of PPI was significantly higher in patients who underwent post-dilatation (46.5% vs 22.9%, *p* = 0.002), and regression analysis demonstrated a significant association between post-dilatation and PPI development. This finding is consistent with previous studies suggesting that the mechanical stress exerted by balloon dilation on septal structures may influence the conduction system [20–22].

In conclusion, the HIGS score demonstrated favorable discriminative performance for predicting both mortality and the requirement for permanent pacemaker implantation after TAVI. However, these findings should be interpreted cautiously because of the retrospective study design, relatively limited event count, and the possibility of residual confounding despite adjustment for major TAVI-specific anatomical and procedural predictors. Although HIGS may represent a promising inflammation-based biomarker reflecting multidimensional hematological and inflammatory alterations, further prospective multicenter studies are required before its routine clinical application can be established.

### a. Limitations of the study

This study has several limitations. First, the study has a retrospective and single-center design. This may introduce potential selection bias in patient inclusion and may limit the generalizability of the findings. In addition, the relatively high mortality rate observed in the present cohort may indicate that the study population represented a particularly high-risk subset of TAVI patients with substantial comorbidity burden and elevated baseline surgical risk. Therefore, caution should be exercised when extrapolating these findings to contemporary lower-risk TAVI populations. The relatively high in-hospital mortality rate observed in this cohort further supports the interpretation that the study population represented a high-risk real-world TAVI group, and this may limit direct comparison with contemporary registries including predominantly lower-risk patients. Nevertheless, the fact that the study is based on real-world clinical data represents an important strength, as it reflects the characteristics of the patient population undergoing TAVI in routine clinical practice.

Second, the sample size and the number of outcome events were limited, particularly for multivariable modeling. The study included 43 PPI events and 34 mortality events, and the number of candidate predictors considered in the regression models may have resulted in a relatively low events-per-variable ratio. Therefore, although the multivariable models were constructed to adjust for clinically relevant confounders and internal bootstrap validation with optimism correction was performed, the possibility of model overfitting and statistical instability cannot be completely excluded. For this reason, the regression models should be interpreted primarily as exploratory risk-adjustment analyses rather than definitive predictive models. Future studies with larger event numbers are required to confirm the stability, calibration, and generalizability of these models.

Third, the hematological parameters used in this study were measured at a single time point prior to the procedure. Considering that inflammatory response is a dynamic process, serial measurements could better reflect temporal changes in inflammatory activity. Future studies evaluating serial hematological measurements during the peri-procedural period may provide a more detailed understanding of the prognostic value of the HIGS score.

Although major TAVI-specific anatomical and procedural predictors of PPI, including membranous septum length, implantation depth, annular calcification grade, LVOT calcification grade, and valve oversizing, were evaluated in the present study, several limitations should be acknowledged. These measurements were obtained retrospectively from available imaging, procedural planning, and procedural documentation rather than through prospective standardized acquisition or independent core laboratory adjudication. In addition, more detailed anatomical variables, such as the precise spatial relationship between the prosthetic valve frame and the conduction system, calcification distribution adjacent to the membranous septum, and operator-dependent implantation technique details, could not be fully captured. Therefore, although the analysis accounted for major established anatomical and procedural predictors, residual confounding cannot be completely excluded. The findings should therefore be considered hypothesis-generating and require confirmation in prospective multicenter studies with standardized imaging protocols.

Finally, the HIGS score is a newly developed composite index derived from routine hematological parameters and requires validation in different patient populations. The weighting coefficients used in the HIGS formula were not derived or optimized within the present TAVI cohort but were based on the previously described HIGS framework and biological plausibility. Although this approach reduces the risk of data-driven model construction within a small sample, it also represents an important limitation because the selected weights may not represent the optimal weighting structure for all TAVI populations. Therefore, the relative contributions of RDW, PDW, and IG% to post-TAVI outcomes should be validated in larger independent cohorts. Future studies may also evaluate alternative weighting strategies, externally derived coefficients, or data-driven optimization approaches to improve the calibration, discrimination, and generalizability of the HIGS score.

## Conclusions

This study demonstrates that the Hematological Inflammatory Global Score (HIGS) may serve as a significant marker for predicting both the need for permanent pacemaker implantation and mortality risk in patients undergoing transcatheter aortic valve implantation. The HIGS score, derived from the combination of RDW, PDW, and IG% values obtained from routine complete blood count parameters, showed strong discriminative performance particularly for predicting permanent pacemaker implantation as well as early mortality after TAVI. These findings suggest that the requirement for permanent pacemaker implantation after TAVI may be influenced not only by mechanical or anatomical factors but also by the patient’s systemic inflammatory status.

From a clinical perspective, the integration of the HIGS score—an easily applicable and cost-effective parameter—into the risk stratification process for TAVI patients may provide important contributions to patient selection, procedural planning, and peri-procedural management. Prospective and multicenter studies with larger sample sizes may further clarify the prognostic role of the HIGS score in conduction system complications and mortality, thereby strengthening the potential clinical application of this biomarker in decision-making processes for patients undergoing TAVI.

## Key points

### a. What is known about the topic?

Permanent pacemaker implantation and early mortality are well-recognized complications following transcatheter aortic valve implantation. Several clinical, electrocardiographic, and procedural factors have been identified as predictors of these outcomes; however, currently used risk models primarily rely on demographic and anatomical variables and often do not incorporate systemic inflammatory biomarkers. Increasing evidence suggests that inflammation plays a significant role in cardiovascular complications and may influence outcomes after interventional procedures such as TAVI.

### b. What does this study add?

This study demonstrates that the Hematological Inflammatory Global Score (HIGS), derived from routine hematological parameters (RDW, PDW, and IG%), is strongly associated with both permanent pacemaker implantation and mortality after TAVI. The HIGS score showed high discriminative performance and provided incremental prognostic value beyond conventional clinical models. These findings suggest that systemic inflammatory status may contribute not only to mortality but also to conduction system complications following TAVI, highlighting the potential role of HIGS as a simple and practical risk stratification tool.

## Data Availability

The data that support the findings of this study are available from the corresponding author upon reasonable request. Due to institutional and ethical regulations, the raw data are not publicly available but can be provided for academic purposes following appropriate approval.

## Declaration of Helsinki

The study and the writing of the article were prepared in accordance with the Declaration of Helsinki.

## Statement on the use of artificial intelligence

The authors used ChatGPT (GPT-5.6 Thinking, OpenAI; accessed July 2026), including its integrated image-generation functionality, solely for two limited purposes [1]: basic English-language editing to improve grammar, wording, and readability without altering the scientific meaning or content of the manuscript; and [2] assistance with the visual design and layout of the Central Illustration based exclusively on the study information, numerical results, terminology, labels, and clinical messages provided and determined by the authors.

Generative artificial intelligence was not used in the conception or design of the study, patient selection, data collection, data extraction, data cleaning, statistical analysis, interpretation of the findings, literature search, reference selection or generation, preparation of the scientific arguments, or formulation of the conclusions. No results, numerical values, statistical estimates, or scientific claims were generated by the AI tool.

All language edits and all components of the Central Illustration were critically reviewed, corrected where necessary, and approved by the authors. No identifiable patient information, raw patient-level data, or confidential clinical information was entered into the AI tool. The authors take full responsibility for the accuracy, integrity, originality, and final content of the manuscript and the Central Illustration.
